# Survey and rapid detection of *Klebsiella pneumoniae* in clinical samples targeting the *rcsA* gene in Beijing, China

**DOI:** 10.3389/fmicb.2015.00519

**Published:** 2015-05-22

**Authors:** Derong Dong, Wei Liu, Huan Li, Yufei Wang, Xinran Li, Dayang Zou, Zhan Yang, Simo Huang, Dongsheng Zhou, Liuyu Huang, Jing Yuan

**Affiliations:** ^1^Institute of Disease Control and Prevention, Academy of Military Medical SciencesBeijing, China; ^2^Department of Laboratory Medicine, The General Hospital of Chinese People's Armed Police ForcesBeijing, China; ^3^State Key Laboratory of Pathogen and Biosecurity, Institute of Microbiology and EpidemiologyBeijing, China

**Keywords:** *Klebsiella pneumoniae*, loop-mediated isothermal amplification, LAMP, rapid detection

## Abstract

*Klebsiella pneumoniae* is a wide-spread nosocomial pathogen. A rapid and sensitive molecular method for the detection of *K. pneumoniae* in clinical samples is needed to guide therapeutic treatment. In this study, we first described a loop-mediated isothermal amplification (LAMP) method for the rapid detection of capsular polysaccharide synthesis regulating gene *rcsA* from *K. pneumoniae*in clinical samples by using two methods including real-time turbidity monitoring and fluorescence detection to assess the reaction. Then dissemination of *K. pneumoniae* strains was investigated from ICU patients in three top hospitals in Beijing, China. The results showed that the detection limit of the LAMP method was 0.115 pg/μl DNA within 60 min under isothermal conditions (61°C), a 100-fold increase in sensitivity compared with conventional PCR. All 30 non- *K. pneumoniae* strains tested were negative for LAMP detection, indicating the high specificity of the LAMP reaction. To evaluate the application of the LAMP assay to clinical diagnosis, of 110 clinical sputum samples collected from ICU patients with clinically suspected multi-resistant infections in China, a total of 32 *K. pneumoniae* isolates were identified for LAMP-based surveillance of *rcs*A. All isolates belonged to nine different *K. pneumoniae* multilocus sequence typing (MLST) groups. Strikingly, of the 32 *K. pneumoniae* strains, 18 contained the *Klebsiella pneumoniae* Carbapenemase (KPC)-encoding gene *bla*_KPC-2_ and had high resistance to β-lactam antibiotics. Moreover, *K. pneumoniae* WJ-64 was discovered to contain *bla*_KPC-2_ and *bla*_NDM-1_genes simultaneously in the isolate. Our data showed the high prevalence of *bla*_KPC-2_ among *K. pneumoniae* and co-occurrence of many resistant genes in the clinical strains signal a rapid and continuing evolution of *K. pneumoniae*. In conclusion, we have developed a rapid and sensitive visual *K. pneumoniae* detection LAMP assay, which could be a useful tool for clinical screening, on-site diagnosis and primary quarantine purposes.

## Introduction

As a Gram-negative bacterium, *Klebsiella pneumoniae* has been identified as a major nosocomial pathogen (Fukigai et al., 2007), which can cause pneumonia, bronchitis, urinary tract and wound infections, especially in infants, diabetics, tumor patients, antibiotic users, and elderly people (Podschun and Ullmann, 1998) in the clinical context. Moreover, antibiotic-resistant *Klebsiella pneumoniae* emerged in recent years has become a serious problem in clinics (Ali Abdel Rahim and Ali Mohamed, 2014). Thus, the rapid and sensitive detection of this pathogen is required if the appropriate therapy is to be administered and outbreaks controlled.

Conventional methods used to detect *K. pneumoniae* based on the phenotypic system include microscopic examination, biochemical identification, and the use of newly developed automatic bacterial identification instruments, such as the VITEK®2 system (Hay et al., 2007). However, they are time-consuming, with low sensitivity, usually requiring several days of incubation. Recently, a number of molecular biological techniques have been used to detect *K. pneumoniae*. PCR based on the 16S-23S internal transcribed spacer was carried out to detect *K. pneumoniae* in infant formula (Liu et al., 2008). Triplex PCR (Jeong et al., 2013) and real-time PCR systems based on SYBR Green (Sun et al., 2010a) have also been used for its specific identification. However, these methods are relatively complex and require specialized, expensive instruments. Moreover, *Taq* DNA polymerase can be inactivated in PCR assays by inhibitors present in crude biological samples (de Franchis et al., 1988).

Therefore, another rapid, simple, and cost-effective assay is required to complement current PCR methods. The loop-mediated isothermal amplification (LAMP) method is a novel nucleic acid detection method, based on auto cycling strand displacement DNA synthesis using *Bst* DNA polymerase under isothermal conditions within 1 h (Notomi et al., 2000). The LAMP method has high specificity because four or six specific primers are used that recognize six or eight different sequences on the target gene. The technique has been widely used in the clinical detection of pathogens, including bacteria (Wei et al., 2008; Hanaki et al., 2011), viruses (Wang et al., 2011), parasites (Chen et al., 2011) and fungi (Sun et al., 2010b), and even in fetal sex determination (Hirayama et al., 2006).

In *K. pneumoniae*, the ability to synthesize large amount of capsular polysaccharide (CPS) is an important correlate of virulence (Goncalves et al., 2014). *RcsA*, a gene specific to *K. pneumoniae* that regulates the synthesis of CPS (Lin et al., 2013), was selected as the target gene in this study. We designed five sets of primers and optimized the LAMP assay to detect *K. pneumoniae*. The specificity and sensitivity of the LAMP method for the detection of *K. pneumoniae* were determined. Finally, clinical isolates of *K. pneumoniae* were identified with LAMP.

## Materials and methods

### Bacterial isolates, identification, MLST typing, antimicrobial susceptibility testing and preparation of templates

A total of 64 bacterial strains were used in this work to develop the LAMP assay (Supplemental Materials). *K. pneumoniae* ATCC BAA-2146 carrying *bla*_NDM−1_ and *K. pneumoniae* ATCC BAA-1705 carrying *bla*_KPC−2_ were used as the positive control. Twenty-one non- *K. pneumoniae* bacterial species maintained in our microorganism center, including common clinical infectious and opportunistic pathogens, were included to evaluate the specificity of the LAMP assay. One hundred and ten clinical sputum samples containing suspected *K. pneumoniae* strains and multi-resistant infections were collected from ICU hospitalized patients with cough or pneumonia in the Wujing hospital, 307 hospital and 301 hospital in Beijing, China. The species identification was carried out using an automated system (Phoenix and BD systems) and matrix-assisted laser desorption ionization time-of-flight mass spectrometry (MALDI-TOF MS). The sequences of 16S ribosomal DNA (rDNA) and *rcs*A were validated by PCR-based sequencing and showed 100% identity with the sequences of previously reported genes.

Seven housekeeper genes including *gap*A, *inf*B, *mdh*, *pgi*, *pho*E, *rop*B, and *tonB* were detected by PCR. The allele number for each gene was assigned to the MLST database (http://bigsdb.web.pasteur.fr/klebsiella/klebsiella.html). A combination of the allelic sequences of the 7 genes yielded the allelic profile. Antimicrobial susceptibility testing was performed by microbroth dilution VITECaccording to the Clinical and Laboratory Standards Institute (CLSI, Performance standards for antimicrobial susceptibility testing; Twenty-third informational supplement CLSI Document M100-S23. Wayne, PA, USA 2013) and Etest strips (bioMérieux) for carbapenems.

The strains were screened for the presence of known MBL and other β-lactamase genes (*bla*_NDM−1_, *bla*_KPC−2_, *bla*_TEM_, *bla*_VIM_, *bla*_IMP_, *bla*_CTX_, *bla*_SIM−1_, *bla*_AIM−1_, and *bla*_OXA−48_) by PCR with primers as reported previously (Poirel et al., 2007; Patzer et al., 2009).

The bacterial strains and clinical samples were cultured in brain heart infusion (BHI) broth at 37°C according to a standard protocol. Genomic DNA was extracted using the Wizard Genomic DNA Purification Kit (Promega Co. USA).

### Primer design

The sequence of the *rcsA* gene (http://www.ncbi.nlm.nih.gov/gene/7946097) was downloaded from NCBI GenBank database and further analyzed by Primer Explorer (Version 4, http://primerexplorer.jp/elamp4.0.0/index.html). Five primer sets were designed (Table 1 and Table S2). To compare the sensitivity of the LAMP and conventional PCR assay, PCR was conducted with the KP-27F3 and KP-27B3 primer pair (Table 1), which amplifies a 176-bp fragment. All primers were synthesized commercially (Sangon Biotech Co., Ltd, Shanghai, China).

**Table 1:** **Primers used for the specific amplification of** ***K. pneumoniae***.

**Primer**	**Type**	**Sequence(5′–3′)**
KP-27F3	Forward outer	GGATATCTGACCAGTCGG
KP-27B3	Backward outer	GGGTTTTGCGTAATGATCTG
KP-27FIP	Forward inner	CGACGTACAGTGTTTCTGCAGTTTTAAAAAACAGGAAATCGTTGAGG
KP-27BIP	Backward inner	CGGCGGTGGTGTTTCTGAATTTTGCGAATAATGCCATTACTTTC
KP-27LB	Loop backward	GAAGACTGTTTCGTGCATGATGA

### LAMP reaction

The LAMP reactions were performed in a final volume of 25 μl containing 12.5 μl reaction mixture, 1 μl *Bst* DNA polymerase, 2 μl template by using the Loopamp DNA Amplification kit (Loopamp DNA Amplification Kit; Eiken Chemical Co., Ltd, Tochigi, Japan) for real-time turbidimeter and another 1 μl calcein/Mn^2+^ solution (Eiken Chemical Co., Ltd) for visual detection. Primers were used at a concentration of 40 pmol for FIP and BIP, 20 pmol for LB and LF, and 5 pmol for F3 and B3. Finally, the reaction mix was overlaid with the protectant to prevent cross-contamination of samples by aerosol and the reactions were performed in the reaction tubes (Eiken Chemical Co. Ltd.) for 60 min at 61°C. During the amplification, the protectant could melted to liquid without disturbing the reaction and solidified as the temperature in the tubes decreased to room time (Patent: ZL201210371448.5 in china).

Two different methods, based on sample turbidity or fluorescence, were used to detect the LAMP products. Real-time changes in turbidity were monitored with spectrophotometric analysis by recording the optical density (650 nm) every 6 s with a Loopamp Realtime Turbidimeter (LA-320c; Eiken Chemical Co., Ltd). For direct visual detection, 1 μl of calcein/Mn^2+^ fluorescent detection reagent was added to the reaction. LAMP amplification results in a green fluorescent emission as a result of magnesium ions forming a complex with calcein. The color change from orange to green when samples are positive is visible to the naked eye under natural light or with the aid of UV light (Tomita et al., 2008). Each experiment was performed at least three times.

### PCR detection

A 25 μl reaction volume was used for all PCRs, with mixtures that contained the following components: 12.5 μl of PCR MasterMix reagents (Tiangen Biotech Co., Ltd, Beijing, China), 9.5 μl of double-distilled water, 1 μM KP-27F3 and KP-27B3 primers, and the same amount of DNA template as was used in the LAMP reaction. The PCR cycling parameters were: initial PCR activation, 95°C for 5 min; amplification, 30 cycles of 95°C for 30 s, 55°C for 30 s, and 72°C for 30 s; final extension, 72°C for 10 min. The products were separated with 1% agarose gel (Amresco) electrophoresis and stained with ethidium bromide. Images were documented with a Gel Doc EQ imaging system (Bio-Rad).

## Results

### Optimization of LAMP assay

**Figure 1:**
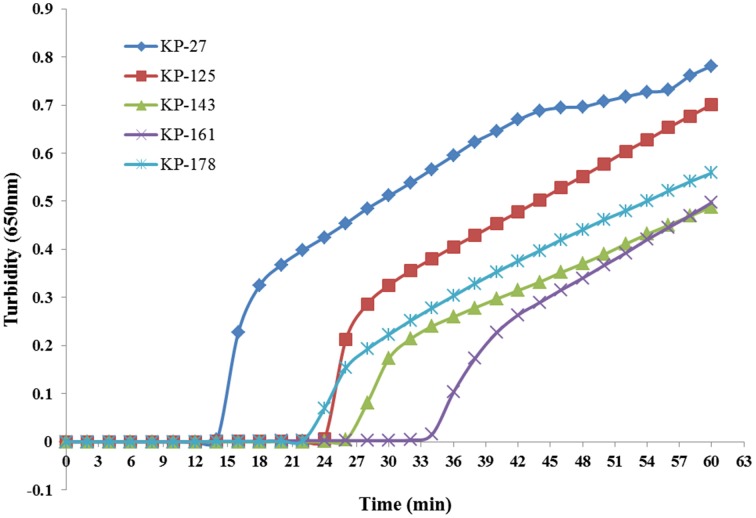
**Five sets of primers were used to amplify the target gene under the same conditions**. Turbidity was monitored every 6 s with a Loopamp Realtime Turbidimeter at 650 nm.

Five sets of primers were initially tested to detect *K. pneumoniae*. All five sets amplified the target sequence under the same reaction conditions. The KP-27 primer set began to amplify the target gene in the shortest time (Figure 1) and was therefore chosen as the optimal primer set for *K. pneumoniae* detection with LAMP (Table 1).

**Figure 2:**
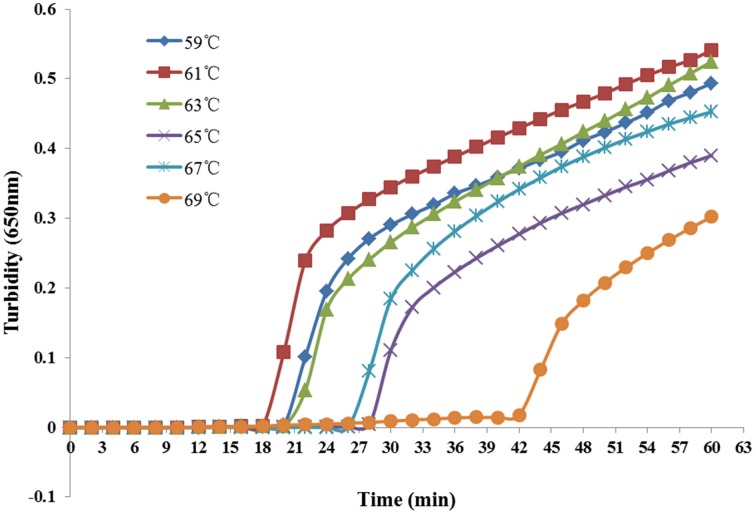
**Different temperatures at which the LAMP reaction detected** ***K. pneumoniae***. Turbidity was monitored every 6 s with a Loopamp Realtime Turbidimeter at 650 nm.

Reaction temperatures ranging from 59°C to 69°C at 2°C intervals were compared for optimal amplification. As shown in Figure 2, the most suitable reaction temperature range was 59–63°C. Finally, we chose 61°C as the optimal reaction temperature.

### Specificity of the LAMP assay

**Figure 3:**
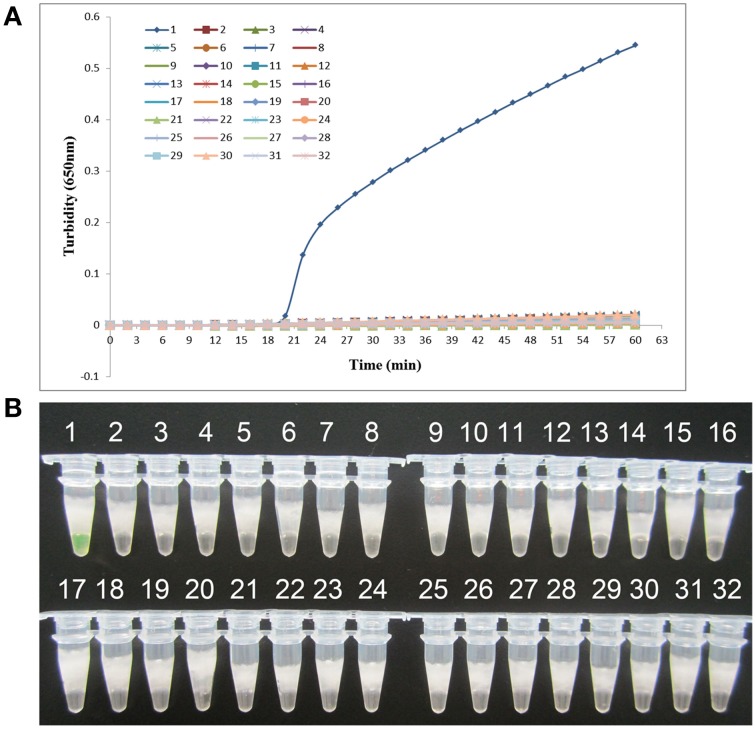
**Specificity of the LAMP reaction in detecting** ***K. pneumoniae*****. (A)** Turbidity was monitored every 6 s using a Loopamp Realtime Turbidimeter at 650 nm. **(B)** The results were visualized by the addition of 1 μl of fluorescent detection reagent to the 25 μl LAMP reaction mixture before the LAMP reaction. Amplification was performed at 61°C for 60 min. 1, positive control (*K. pneumoniae* ATCC BAA-2146); 2, negative control (double-distilled water); 3, *Klebsiella oxytoca* ATCC 700324; 4, *Klebsiella rhinoscleromatis* CMCC 46111; 5, *Citrobacter freundii* CMCC 48001; 6, *Enterobacter aerogenes* ATCC 13048; 7, *Enterobacter cloacae* ATCC 13047; 8, *Proteus mirabilis* CMCC 49005; 9, *Proteus vulgaris* CMCC 49027; 10, *Serratia marcescens* ATCC 14756; 11, *Morganella morganii* ATCC 25830; 12, *Streptococcus pneumoniae* 112-07; 13, *Mycobacterium tuberculosis* 005; 14, *Pseudomonas aeruginosa* D104; 15, *Haemophilus influenza* ATCC 49247; 16, *Yersinia enterocolitica* 027; 17, *Yersinia pestis* 2638; 18, *Bacillus tularense* 3450; 19, *Vibrio cholera* 3802; 20, *Salmonella aberdeen* 9264; 21, *Neisseria meningitides* CMCC 29022; 22, *Staphylococcus aureus* 2740; 23, *Pseudomonas pseudomallei* 029; 24, *Salmonella typhimurium* 4030; 25, *Corynebacterium diphtheriae* CMCC 38001; 26, *Bacillus megatherium* 4623; 27, *Stenotrophomonas maltophilia* K279a; 28, *Legionella pneumophila* 9135; 29, *Acinetobacter baumannii* 12101; 30, enteroinvasive *E. coli* 44825; 31, enterotoxigenic *E. coli* 44824; 32, enteropathogenic *E. coli* 2348.

*K. pneumoniae* ATCC BAA-2146 was used as the positive control and double-distilled water as the negative control when evaluating the specificity of LAMP for the detection of *K. pneumoniae*. Twenty-one other non- *K. pneumoniae* bacterial strains (Supplemental Materials) were also tested. As shown in Figure 3, both methods of analysis positively identified the *K. pneumoniae*. All other strains, including the blank control, tested negative, indicating that the LAMP assay was specific to *K. pneumoniae*.

### Sensitivity of the LAMP assay vs. PCR for K. pneumoniae detection

**Figure 4:**
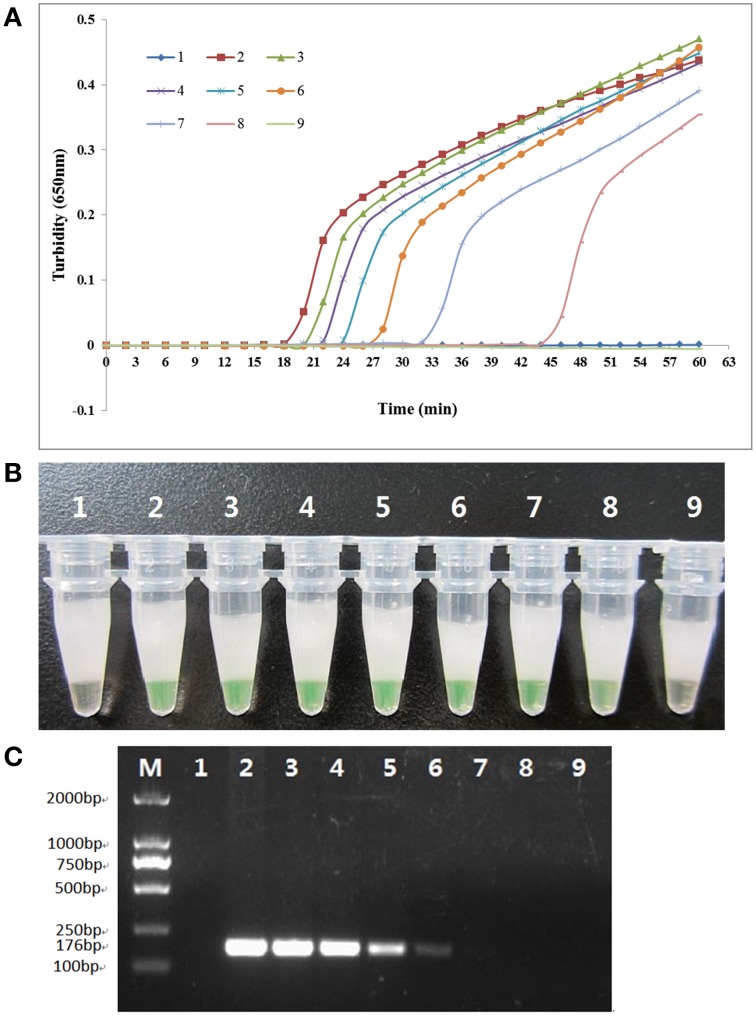
**Comparison of the sensitivities of the LAMP reaction and conventional PCR in detecting the** ***rcsA*****gene of** ***K. pneumoniae***. Pure genomic DNA extracted from *K. pneumoniae* ATCC BAA-2146 was serially diluted 10-fold. **(A)** Turbidity was monitored every 6 s with a Loopamp Realtime Turbidimeter at 650 nm. **(B)** The reaction result was detected visually by the addition of 1 μl of fluorescent detection reagent to the 25 μl LAMP reaction mixture before the LAMP reaction. **(C)** PCR products were separated by 2% agarose gel electrophoresis and stained with ethidium bromide. Amplification was performed at 61°C for 60 min. 1, negative control (double-distilled water); 2, 115.0 ng/μl; 3, 11.5 ng/μl; 4, 1.15 ng/μl; 5, 115.0 pg/μl; 6, 11.5 pg/μl; 7, 1.15 pg/μl; 8, 0.115 pg/μl; 9, 0.0115 pg/μl.

To compare the detection limit of LAMP using either real-time turbidity measurements or visual color change with traditional PCR, pure genomic DNA was extracted from *K. pneumoniae* ATCC BAA-2146 and subjected to serial 10-fold dilution, from 115.0 ng/μl to 0.0115 pg/μl. As shown in Figure 4, the detection limit of the real-time turbidity and visual detection was both 0.115 pg/μl, which was 100-fold more sensitive than traditional PCR assay.

### Dissemination of K. pneumoniae in clinic

**Figure 5:**
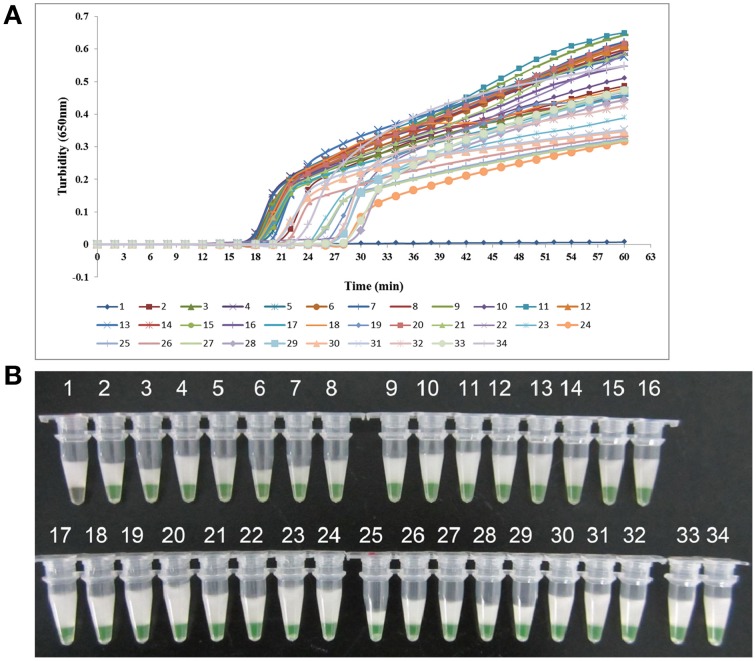
**LAMP results for** ***K. pneumoniae*** **isolates from clinical samples. (A)** Turbidity was monitored every 6 s using a Loopamp Realtime Turbidimeter at 650 nm. **(B)** The results were visualized by the addition of 1 μl of fluorescent detection reagent to the 25 μl LAMP reaction mixture before the LAMP reaction. 1, Negative control (double-distilled water); 2, positive control (*K. pneumoniae* ATCC BAA-2146); 3, *K. pneumoniae* WJ-48; 4, *K. pneumoniae* WJ-50; 5, *K. pneumoniae* WJ-51; 6, *K. pneumoniae* WJ-52; 7, *K. pneumoniae* WJ-53; 8, *K. pneumoniae* WJ-57; 9, *K. pneumoniae* WJ-58; 10, *K. pneumoniae* WJ-60; 11, *K. pneumoniae* WJ-61; 12, *K. pneumoniae* WJ-64; 13, *K. pneumoniae* WJ-65; 14, *K. pneumoniae* WJ-66; 15, *K. pneumoniae* WJ-68; 16, *K. pneumoniae* 301-052; 17, *K. pneumoniae* 301-207; 18, *K. pneumoniae* 301-263; 19, *K. pneumoniae* 301-432; 20, *K. pneumoniae* 301-416; 21, *K. pneumoniae* 301-323; 22, *K. pneumoniae* 301-365; 23, *K. pneumoniae* 301-282; 24, *K. pneumoniae* 301-406; 25, *K. pneumoniae* 301-158; 26, *K. pneumoniae* 307-206; 27, *K. pneumoniae* 307-082; 28, *K. pneumoniae* 307-429; 29, *K. pneumoniae* 307-095; 30, *K. pneumoniae* 307-003; 31, *K. pneumoniae* 307-030; 32, *K. pneumoniae* 307-194; 33, *K. pneumoniae* 307-356; 34, *K. pneumoniae* 307-235.

One hundred and ten clinical sputum samples were collected for LAMP-based surveillance of *K. pneumoniae* from ICU patients with clinically suspected multi-resistant infections in three top hospitals of Beijing. Ten sputum samples from healthy people were collected as controls. Both LAMP and PCR assay were involved to analyze the clinical samples. As shown in Figure 5, of the 110 clinical samples, LAMP detected 32 positive samples while 25 were detected by PCR. Then, 32 *K. pneumoniae* strains were successfully cultured from these positive samples. The healthy control samples all tested negative in each of the assays. The sequence analysis of the *rcs*A genes from *K. pneumoniae* isolates confirmed conservation with the nucleotide sequences of reported gene.

MLST analysis showed that the 32 *K. pneumoniae* strains belonged to different sequence type (ST) including ST11, ST21, ST30, ST37, ST40, ST84, ST104, ST147 or ST322, respectively (Supplemental Materials). To further characterize the clinical *K. pneumoniae* isolates, PCR screening of MBL and other β-lactamase genes was performed and antimicrobial susceptibility tested, the results showed a co-occurrence of resistance genes in most of the *K. pneumoniae* clinical isolates. The positive PCR products were sequenced and the result showed 100% identities with previously reported genes. It is interesting to note that 18 of the 32 *K. pneumoniae* strains contain the *bla*_KPC−2_ gene, resulting in increased resistance to β-lactams antibiotics (Supplemental Materials). Moreover, the isolate named *K. pneumoniae* WJ-64 simultaneously contained *bla*_KPC−2_ and *bla*_NDM−1_which has emerged in recent years and attracted wide attention, presented increased resistance to all β-lactams (MIC > 128μg/ml for meropenem and imipenem), cephalosporins and aminoglycosides, and was only susceptible to tigecycline. The isolate was also positive for imipenem-EDTA double-disk synergy test (DDST) and modified Hodge test (MHT).

## Discussion

*Klebsiella pneumoniae* is a widespread nosocomial pathogen and the most significant member of the genus *Klebsiella* in the family Enterobacteriaceae. Researchers have confirmed that the bacteremia caused by *K. pneumoniae* can greatly increase in-patient mortality (Chetcuti Zammit et al., 2014). In the past two decades, *K. pneumoniae* has surpassed *Escherichia coli* as the predominant species isolated from patients with pyogenic liver abscess globally (Liu et al., 2013). With the acquisition of antibiotic-resistant genes (such as those encoding *bla*_KPC−2_, *bla*_NDM−1_, and *bla*_TEM_), it is increasingly difficult to cure carbapenem-resistant *K. pneumoniae* (de Sanctis et al., 2014). Therefore, the early diagnosis of this pathogen has become increasingly important.

To meet this challenge, we have established a novel detection assay using the LAMP method, which can be completed within 60 min. We selected the capsular polysaccharide synthesis regulating gene *rcsA* as the target gene because it is more stable. Other specific genes of *K. pneumoniae*, such as *phoE* and *tyrB*, always have some mutants in their sequences. As far as we know, this is the first study to apply the LAMP method to the detection of *K. pneumoniae*. The results of sensitivity and specificity experiments show that the LAMP assay is 100-fold more sensitive than the conventional PCR assay, and is specific for *K. pneumoniae*. Furthermore, the LAMP reaction requires only a constant-temperature environment, so a temperature-controlled water bath that can be stably heated is sufficient, whereas PCR must be performed under temperature-cycling conditions. Importantly, Kaneko found that the LAMP reaction is not susceptible to the influence of the different components often present in clinical samples. Thus, the purification of DNA from a sample is not necessary (Kaneko et al., 2007). Although the LAMP assay has a complex amplification principle, it is rapid, easy to operate, highly sensitive and specific. Therefore, it is appropriate for the detection of *K. pneumoniae*, especially for routine diagnosis and infection control purposes.

A drawback of the LAMP method is that it has a relatively high rate of false-positive results. This is because the amplification efficiency of the LAMP assay is extremely high, and 20 μg of specific DNA can be synthesized in a 25 μl reaction mixture within 60 min (Mori et al., 2001). Strict spatial separation between the reagent preparation and the performance of the test is also very important in avoiding contamination. In this study, a sealing agent was added to the reaction tube after the reaction reagents were prepared to prevent the spread of the amplification products, and proved useful in precluding contamination.

Application of LAMP assay to samples taken from hospital admissions of cough or pneumonia indicated that *K. pneumoniae* was prevalent with nearly 30% of samples tested positive. Moreover, there was a high prevalence of *bla*_KPC−2_ among *K. pneumoniae*. Diverse MLST types of *K. pneumoniae* carrying *bla*_KPC−2_ and co-occurrence of many resistance genes in the clinical strains signal a rapid and continuing evolution of *K. pneumoniae* resulting from their wide spread in clinical infections, and there would be a lot of difficulties to control.

In conclusion, a specific, sensitive, rapid, and effective method for the detection of *K. pneumoniae* with the loop-mediated isothermal amplification method was established. We anticipate its routine use in hospital and point-of-care testing regimes, particularly for rapid clinical diagnoses where time and resources are limited.

### Conflict of interest statement

The authors declare that the research was conducted in the absence of any commercial or financial relationships that could be construed as a potential conflict of interest.
