# Supplementary material for: Survey and rapid detection of Klebsiella pneumoniae in clinical samples targeting the rcsA gene in Beijing, China
Source: Front Microbiol. 2015 May 22;6:519. doi: 10.3389/fmicb.2015.00519 (PMC4440914; doi:10.3389/fmicb.2015.00519)
Supplement: Supplementary file 1 [file Table1.DOCX]

**Supplementary Materials**

**Table 1:** Bacterial strains used in the study

| Strains | Source |
| --- | --- |
| *K. pneumoniae* ATCC BAA-2146 | Our microorganism center |
| *K. pneumoniae* ATCC BAA-1705 | Our microorganism center |
| *K. pneumoniae* WJ-48 | Clinical isolate |
| *K. pneumoniae* WJ-50 | Clinical isolate |
| *K. pneumoniae* WJ-51 | Clinical isolate |
| *K. pneumoniae* WJ-52 | Clinical isolate |
| *K. pneumoniae* WJ-53 | Clinical isolate |
| *K. pneumoniae* WJ-57 | Clinical isolate |
| *K. pneumoniae* WJ-58 | Clinical isolate |
| *K. pneumoniae* WJ-60 | Clinical isolate |
| *K. pneumoniae* WJ-61 | Clinical isolate |
| *K. pneumoniae* WJ-64 | Clinical isolate |
| *K. pneumoniae* WJ-65 | Clinical isolate |
| *K. pneumoniae* WJ-66 | Clinical isolate |
| *K. pneumoniae* WJ-68 | Clinical isolate |
| *K. pneumoniae* 301-052 | Clinical isolate |
| *K. pneumoniae* 301-207 | Clinical isolate |
| *K. pneumoniae* 301-263 | Clinical isolate |
| *K. pneumoniae* 301-432 | Clinical isolate |
| *K. pneumoniae* 301-416 | Clinical isolate |
| *K. pneumoniae* 301-323 | Clinical isolate |
| *K. pneumoniae* 301-365 | Clinical isolate |
| *K. pneumoniae* 301-282 | Clinical isolate |
| *K. pneumoniae* 301-406 | Clinical isolate |
| *K. pneumoniae* 301-158 | Clinical isolate |
| *K. pneumoniae* 307-206 | Clinical isolate |
| *K. pneumoniae* 307-082 | Clinical isolate |
| *K. pneumoniae* 307-429 | Clinical isolate |
| *K. pneumoniae* 307-095 | Clinical isolate |
| *K. pneumoniae* 307-003 | Clinical isolate |
| *K. pneumoniae* 307-030 | Clinical isolate |
| *K. pneumoniae* 307-194 | Clinical isolate |
| *K. pneumoniae* 307-356 | Clinical isolate |
| *K. pneumoniae* 307-235 | Clinical isolate |
| *Klebsiella oxytoca* ATCC 700324 | Our microorganism center |
| *Klebsiella rhinoscleromatis* CMCC 46111 | Our microorganism center |
| *Citrobacter freundii* CMCC 48001 | Our microorganism center |
| *Enterobacter aerogenes* ATCC 13048 | Our microorganism center |
| *Enterobacter cloacae* ATCC 13047 | Our microorganism center |
| *Proteus mirabilis* CMCC 49005 | Our microorganism center |
| *Proteus vulgaris* CMCC 49027 | Our microorganism center |
| *Serratia marcescens* ATCC 14756 | Our microorganism center |
| *Morganella morganii* ATCC 25830 | Our microorganism center |
| *Streptococcus pneumoniae* 112-07 | Our microorganism center |
| *Mycobacterium tuberculosis* 005 | Our microorganism center |
| *Pseudomonas aeruginosa* D104 | Our microorganism center |
| *Haemophilus influenza* ATCC 49247 | Our microorganism center |
| *Yersinia enterocolitica* 027 | Our microorganism center |
| *Yersinia pestis* 2638 | Our microorganism center |
| *Bacillus tularense* 3450 | Our microorganism center |
| *Vibrio cholera* 3802 | Our microorganism center |
| *Salmonella aberdeen* 9264 | Our microorganism center |
| *Neisseria meningitides* CMCC 29022 | Our microorganism center |
| *Staphylococcus aureus* 2740 | Our microorganism center |
| *Pseudomonas psedomallei* 029 | Our microorganism center |
| *Salmonella typhimurium* 4030 | Our microorganism center |
| *Corynebacterium diphtheria* CMCC 38001 | Our microorganism center |
| *Bacillus megatherium* 4623 | Our microorganism center |
| *Stenotrophomonas maltophilia* K279a | Our microorganism center |
| *Legionella pneumophila* 9135 | Our microorganism center |
| *Acinetobacter baumannii* 12101 | Our microorganism center |
| Enteroniavasive *E. coli* 44825 | Our microorganism center |
| Enterotoxigenic *E. coli* 44824 | Our microorganism center |
| Enteropathogenic *E. coli* 2348 | Our microorganism center |
