# Supplementary material for: Survey and rapid detection of Klebsiella pneumoniae in clinical samples targeting the rcsA gene in Beijing, China
Source: Front Microbiol. 2015 May 22;6:519. doi: 10.3389/fmicb.2015.00519 (PMC4440914; doi:10.3389/fmicb.2015.00519)
Supplement: Supplementary file 2 [file Table2.DOCX]

**Supplementary Materials**

**Table 2:** The rest 4 sets of primers used in this study

| **Primer Set** | **Primer** | **Sequence(5'-3')** |
| --- | --- | --- |
| KP-143 | KP-143F3 | TCTTAAATACAAAAACACCAGTG |
|  | KP-143B3 | ACCTGCTTATTATGCGTTTG |
|  | KP-143FIP | CCACATTTGCAGCATATTTGATTCTTTTTAGGGCAGTTAACTTTACCG |
|  | KP-143BIP | GGCATGGTACTTCGCAAATCTTTTCTTTATGCGACGATACCGTC |
|  | KP-143LB | CAACGCAAATGAACATCAAAGCG |
| KP-161 | KP-161F3 | AAACACCAGTGTAGGGCA |
|  | KP-161B3 | CCTGCTTATTATGCGTTTGT |
|  | KP-161FIP | GCCATCCACATTTGCAGCATTTTAACTTTACCGACATTGTCAC |
|  | KP-161BIP | GGCATGGTACTTCGCAAATCTTTTTTATGCGACGATACCGTC |
|  | KP-161LB | CAACGCAAATGAACATCAAAGCG |
| KP-125 | KP-125F3 | TGCTAATCAGTTCAAAATCGA |
|  | KP-125B3 | GATGTTCATTTGCGTTGAGA |
|  | KP-125FIP | CTGCCCTACACTGGTGTTTTTTTAAAAGACCTTGATGTTATTCTGG |
|  | KP-125BIP | TAACTTTACCGACATTGTCACTGAGTTTTTGCGAAGTACCATGCC |
|  | KP-125LB | TCAAATATGCTGCAAATGTGGATGG |
| KP-178 | KP-178F3 | ACATTGTCACTGAGTAAAACAG |
|  | KP-178B3 | GAATACCGGAGGTGATGTT |
|  | KP-178FIP | TCATTTGCGTTGAGATTTGCGAATTTTAATCAAATATGCTGCAAATGTG |
|  | KP-178BIP | CGAAGACGGTATCGTCGCATTTTCGATATGATAAATCACCTGCTTA |
|  | KP-178LF | TACCATGCCCGGCCATC |
