# Supplementary material for: Survey and rapid detection of Klebsiella pneumoniae in clinical samples targeting the rcsA gene in Beijing, China
Source: Front Microbiol. 2015 May 22;6:519. doi: 10.3389/fmicb.2015.00519 (PMC4440914; doi:10.3389/fmicb.2015.00519)
Supplement: Supplementary file 3 [file Table3.DOCX]

**Supplementary Materials**

**Table 3:** The ST types of *K. pneumoniae* isolates

| ST Type | Isolates |
| --- | --- |
| ST11 | WJ-50, WJ-51, WJ-58, WJ-64, WJ-65, 301-207, 301-432, 301-282, 307-082, 307-429, 307-194, 307-235 |
| ST21 | WJ-48, WJ-68, WJ-66 |
| ST30 | WJ-60, 301-416, 301-323, 301-406 |
| ST37 | 307-003, WJ-61, 301-365,307-206 |
| ST40 | 307-095 |
| ST84 | 307-030, 307-356 |
| ST104 | WJ-53, 301-158 |
| ST147 | WJ-57, 301-052 |
| ST322 | WJ-52, 301-263 |
