# Supplementary material for: Survey and rapid detection of Klebsiella pneumoniae in clinical samples targeting the rcsA gene in Beijing, China
Source: Front Microbiol. 2015 May 22;6:519. doi: 10.3389/fmicb.2015.00519 (PMC4440914; doi:10.3389/fmicb.2015.00519)
Supplement: Supplementary file 4 [file Table4.DOCX]

**Supplementary Materials**

**Table 4:** The antibiotic susceptibility of *K. pneumoniae* containing *bla*_KPC-2_ (μg/ml).

| Isolate No. | IMP | MEM | CTX | CTZ | FEP | GEN | ATM | AMP | TZP | TOB | COL | TGC |
| --- | --- | --- | --- | --- | --- | --- | --- | --- | --- | --- | --- | --- |
| WJ-50 | 4 | 8 | ≥32 | ≥64 | ≥64 | 8 | ≥64 | ≥32 | ≥128 | 16 | 0.125 | 0.5 |
| WJ-51 | 4 | 8 | ≥32 | ≥64 | ≥64 | 8 | ≥64 | ≥32 | ≥128 | 16 | 0.125 | 0.5 |
| WJ-52 | 16 | ≥16 | ≥64 | ≥32 | ≥64 | 4 | ≥64 | ≥32 | ≥128 | 8 | 0.5 | 0.5 |
| WJ-58 | ≥16 | 16 | ≥32 | ≥64 | ≥64 | 16 | 128 | ≥32 | ≥128 | 16 | 0.125 | 1 |
| WJ-64 | ≥128 | ≥128 | ≥256 | ≥256 | ≥256 | ≥32 | ≥256 | ≥32 | ≥256 | ≥16 | 2 | 2 |
| WJ-66 | 16 | 8 | ≥128 | ≥64 | ≥32 | 16 | 32 | ≥32 | ≥256 | 8 | 1 | 1 |
| 301-052 | 8 | 4 | ≥64 | ≥64 | ≥64 | 8 | 32 | ≥32 | ≥128 | 8 | 0.125 | 0.5 |
| 301-207 | 8 | 16 | ≥64 | ≥64 | ≥32 | 8 | 16 | ≥32 | ≥64 | 8 | 0.5 | 0.5 |
| 301-263 | ≥16 | ≥16 | ≥64 | ≥64 | ≥64 | 4 | 16 | ≥32 | ≥128 | 8 | 0.5 | 0.25 |
| 301-323 | 16 | 8 | ≥64 | ≥64 | ≥64 | 2 | ≥64 | ≥32 | 64 | 16 | 0.5 | 1 |
| 301-282 | 4 | 16 | ≥32 | ≥64 | ≥32 | 16 | 32 | ≥32 | ≥128 | 16 | 1 | 1 |
| 301-158 | ≥16 | ≥16 | ≥64 | ≥32 | ≥64 | 16 | 32 | ≥32 | ≥64 | ≥16 | 0.25 | 1 |
| 307-082 | 16 | 16 | ≥64 | ≥64 | ≥32 | 8 | 16 | ≥32 | ≥64 | ≥16 | 0.25 | 1 |
| 307-429 | 16 | 16 | ≥64 | ≥64 | ≥64 | 8 | ≥64 | ≥32 | ≥128 | 16 | 0.5 | 0.5 |
| 307-003 | ≥8 | 16 | ≥32 | ≥64 | ≥64 | 4 | ≥64 | ≥32 | ≥128 | 8 | 1 | 1 |
| 307-030 | 16 | 8 | ≥64 | ≥32 | ≥32 | 16 | 64 | ≥32 | 64 | 8 | 0.5 | 0.5 |
| 307-356 | 8 | 4 | ≥64 | ≥64 | ≥64 | 16 | 32 | ≥32 | ≥128 | 16 | 0.5 | 0.5 |
| 307-235 | 16 | 8 | ≥64 | ≥64 | ≥64 | 4 | 32 | ≥32 | ≥128 | 8 | 0.5 | 1 |

IMP, imipenem; MEM, meropenem; CTX, cefotaxime; CTZ, ceftazidime; FEP, cefepime; GEN, gentamicin; ATM, aztreonam; AMP, ampicillin; TZP, piperacillin-tazobactam; TOB, Tobramycin; COL, colisin; TGC, tigecycline.
